# Resolution of structure of PIP5K1A reveals molecular mechanism for its regulation by dimerization and dishevelled

**DOI:** 10.1038/ncomms9205

**Published:** 2015-09-14

**Authors:** Jian Hu, Qianying Yuan, Xue Kang, Yuanbo Qin, Lin Li, Ya Ha, Dianqing Wu

**Affiliations:** 1 Department of Pharmacology, Yale School of Medicine, New Haven, Connecticut 06520, USA; 2 Vascular Biology and Therapeutic Program, Yale School of Medicine, New Haven, Connecticut 06520 USA; 3 State Key Laboratory of Molecular Biology, Institute of Biochemistry and Cell Biology, Shanghai Institutes for Biological Sciences, Chinese Academy of Sciences, Shanghai 200031, China

## Abstract

Type I phosphatidylinositol phosphate kinase (PIP5K1) phosphorylates the head group of phosphatidylinositol 4-phosphate (PtdIns4P) to generate PtdIns4,5P_2_, which plays important roles in a wide range of cellular functions including Wnt signalling. However, the lack of its structural information has hindered the understanding of its regulation. Here we report the crystal structure of the catalytic domain of zebrafish PIP5K1A at 3.3 Å resolution. This molecule forms a side-to-side dimer. Mutagenesis study of PIP5K1A reveals two adjacent interfaces for the dimerization and interaction with the DIX domain of the Wnt signalling molecule dishevelled. Although these interfaces are located distally to the catalytic/substrate-binding site, binding to these interfaces either through dimerization or the interaction with DIX stimulates PIP5K1 catalytic activity. DIX binding additionally enhances PIP5K1 substrate binding. Thus, this study elucidates regulatory mechanisms for this lipid kinase and provides a paradigm for the understanding of PIP5K1 regulation by their interacting molecules.

## Abstract (web summary)

Type I phosphatidylinositol phosphate kinase is an important component of many cellular pathways, including Wnt signalling. Here the authors report the crystal structure of the zebrafish protein along with *in vitro* assays that help to elucidate the regulation and function of this kinase.

## Graphical abstract

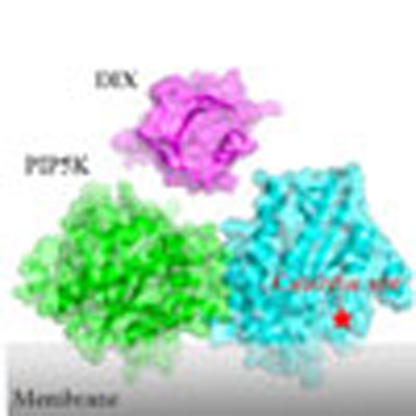

Although sphosphoinositides constitute only a small fraction of the eukaryotic cell membrane, they are essential for a wide range of cellular activities, including signal transduction and membrane trafficking^1^,^2^. The prototypic phosphoinositide, phosphatidylinositol 4,5-bisphosphate (PtdIns4,5P_2_), is the substrate for phospholipase C, whose action produces two key second messengers, inositol 1,4,5-trisphosphate and diacylglycerol, in calcium signalling and protein kinase activation. PtdIns4,5P_2_ can also be converted by phosphatidylinositol 3-kinase (PI3K) to phosphatidylinositol 3,4,5-trisphosphate, which is important in signal transduction and cancer biology^3^. PtdIns4,5P_2_ itself regulates the functions of many proteins by recruiting them to specific membrane compartments, and by directly modulating their activities^4^. PtdIns4,5P_2_ can be synthesized from phosphatidylinositol 4-phosphate (PtdIns4P) and phosphatidylinositol 5-phosphate by type I and type II phosphatidylinositol phosphate kinases (PIPKs), respectively^4^. The reaction catalysed by the type I PIPK, or phosphatidylinositol 4-phosphate 5-kinase (PIP5K1), is the major synthetic route for PtdIns4,5P_2_ in most cells^5^.

PtdIns4,5P_2_, whose production can be strongly stimulated by WNT3A in cells, has an important role in Wnt signal transduction. WNT3A belongs to the Wnt family of secretory glycoproteins that are involved in a wide range of biological and pathophysiological processes, including embryonic development, organogenesis, tissue homeostasis, stem cell biology, lipid and glucose metabolism and tumorigenesis^6^,^7^. WNT3A initiates its signal transduction by binding to two cell-surface coreceptors, frizzled and low-density lipoprotein-related protein (LRP) 5/6, leading to the LRP phosphorylation and subsequent recruitment of Axin to the phosphorylated LRP. These steps eventually result in stabilization and accumulation of a multifunctional protein β-catenin^8^,^9^,^10^. Wnt3a-induced LRP phosphorylation requires the formation of LRP signalosomes^11^, and PtdIns4,5P_2_ is involved in LRP signalosome formation: it recruits clathrin-AP2 to LRP6 to form stable protein clusters on the cell surface^12^ and recruits Axin to the membrane by the PtdIns4,5P_2_-binding protein Amer1/wildtypeX (ref. 13). The enhanced PtdIns4,5P_2_ production on Wnt3a stimulation appears to result from a direct interaction between the lipid kinases and dishevelled (DVL), which is recruited to Fz on ligand binding: the N-terminal DIX domain of DVL binds PIP5K1 and enhances its catalytic activity, whereas the C-terminal DEP domain of DVL binds and activates phosphatidylinositol 4-kinase^14^,^15^.

The three-dimensional structure of PIP5K1, which consists of three isoforms (A, B and C)^16^,^17^,^18^,^19^, was previously unknown. Although the crystal structure of a homologous type II PIPK, human PIP4K2B (also known as PIP4Kβ), was resolved many years ago^20^, PIP4K2 is not involved in Wnt signalling^14^. To help elucidating the mechanism for PIP5K1 regulation by DVL, we determine the X-ray structure of the kinase domain of zebrafish PIP5K1A. Comparison with the known structure of PIP4K2B yields important insights into the unique mechanism of interfacial catalysis for this important family of enzymes. More importantly, the new crystal structure enables us to delineate two adjacent interfaces for dimerization and interaction with DVL that are unique to the PIP5K1 kinases and unravel the importance of these interfaces in regulation of the kinase enzymatic activity by dimerization and interaction with DVL.

## Results

### The tertiary fold of type I PIPK

**Figure 1:**
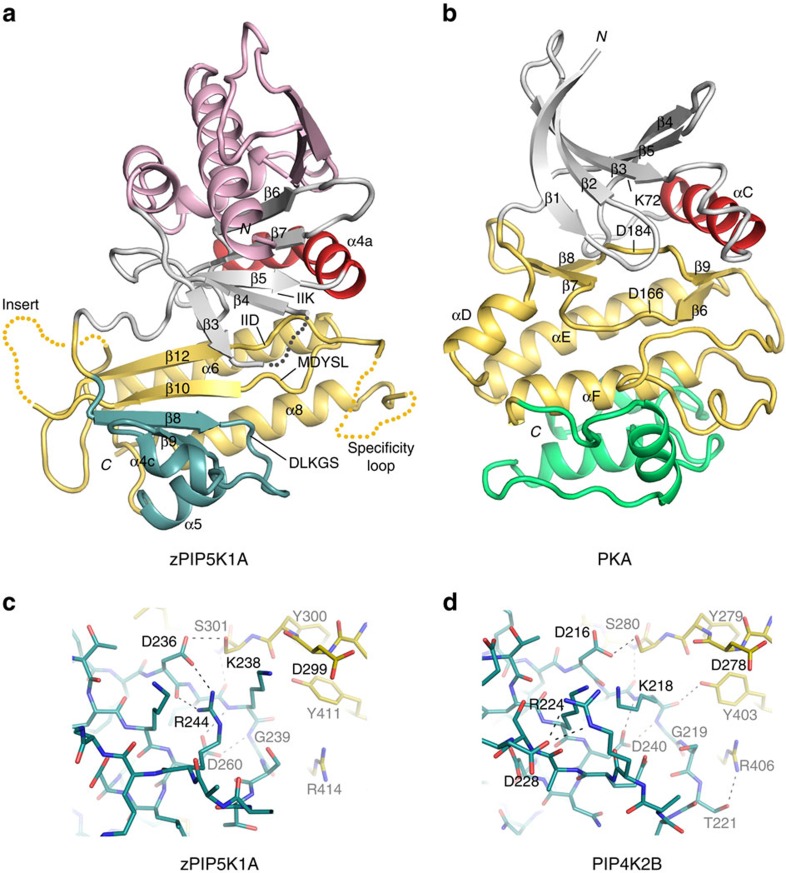
PIP5K1A structure. (**a**,**b**) Crystal structure of zebrafish PIP5K1A kinase catalytic domain and its comparison with that of PKA. The N- and C-terminal segments outside the kinase core domain are omitted to better illustrate the protein kinase fold. In both panels, the N-lobe is coloured grey and the C-lobe is yellow. The ‘C helix’ is highlighted in red. The substructure harbouring the lipid kinase’s DLKGS motif is highlighted in blue. The N-terminal secondary structures found only in phosphatidylinositol phosphate kinases are shown in pink. The structure C-terminal to PKA’s αF, missing in the phosphatidylinositol phosphate kinases, is coloured green. The disordered regions are indicated by dotted lines. (**c**,**d**) The DLKGS motifs of zPIP5K1A and PIP4K2B. The substructure (blue) harbouring the DLKGS motif reveals differences between zPIP5K1A (**c**) PIP4K2B (**d**). The C-lobe of the kinase is coloured yellow. Dashed lines represent potential hydrogen bonds.

The structure of zebrafish PIP5K1A kinase domain was determined by molecular replacement using human PIP4K2B as the search model (Protein Data Bank (PDB) accession code 1BO1; Rao *et al*.^20^), and refined to 3.3 Å resolution (Table 1)(Supplementary Fig. 1). These two PIPKs are homologous in amino-acid sequence (31% identical), and their backbone C_α_s can be superimposed with a root-mean-squared deviation of 1.26 Å. Besides a few of the residues at the two termini of the protein, three regions of the kinase are disordered in the crystal (dotted line in Fig. 1a): a short turn (residues 154–156 of PIP5K) between β3 and β4 (secondary structure elements named according to Rao *et al*.^20^; Supplementary Fig. 2), which corresponds to the glycine-rich loop in the N-lobe of protein kinases; a highly divergent sequence after β10 called ‘insert’ (residues 310–356; the disordered region includes helix α7, which is visible in PIP4K2B); and the ‘specificity loop’ (residues 386–401), which corresponds to the activation segment of protein kinases^21^.

Like PIP4K2B (Rao *et al*.^20^), the fold of PIP5K1A is similar to those of protein kinases, including PKA (PDB accession number 1ATP)^22^ (Fig. 1b). The N-terminal lobe of the lipid kinase contains three α-helices (α1–3) and two β-strands (β1 and β2) that are unique to the PIPKs (pink in Fig. 1a). This additional domain is stacked on top of a five-stranded β-sheet (β3–7; β1 and β2 are continuous with the sheet) that is equivalent to the antiparallel sheet (β1–5) in protein kinase A (PKA; grey in Fig. 1b). In PIP4K2B and PIP5K1A, the helix (α4) connecting β5 and β6 corresponds topologically to the ‘C helix’ of PKA, but is bent in the middle (red in Fig. 1a). The helices downstream of αF in PKA are missing in the PIPKs (green in Fig. 1b). The locations of Lys-171 (from the conserved ‘IIK’ motif), Asp-299 (MDYSL) and Asp-378 (IID) in the three-dimensional structure of PIP5K1A suggest that they are functionally equivalent to Lys-72, Asp-166 and Asp-184 of PKA, which participate in ATP binding and catalysis.

Immediately following the linker between the N- and C-terminal lobes, the PIPKs contain a subdomain that contains the ‘DLKGS’ sequence motif and is absent in protein kinases (cyan in Fig. 1a). Here the structure is most different between PIP5K1A and PIP4K2B (Fig. 1c,d). In PIP5K1A, the turn between β8 and α4c is compact. The side chain of Arg-244 is potentially hydrogen bonded to the main chain carbonyl oxygen and side chain carboxylate group of Asp-236, and pushes the side chain of Lys-238 into the active site. In PIP4K2B, the turn adopts a more relaxed fold, and appears to be stabilized by hydrogen bonds with the conserved Tyr-403 (PIP4K2B numbering) and Arg-406 from β8 in the C-lobe. The side chains of Arg-224 and Lys-218 (corresponding to Arg-244 and Lys-238 in PIP5K1A) both swing to the left: Arg-224 now forms a salt bridge with a different aspartate (Asp-228) that is four residues downstream. The ability of the turn to acquire this alternative fold is probably influenced by Asp-228. Since all of PIP4K2s have either an aspartate or glutamate at position 228, whereas other residue types, most often a positively charged arginine or lysine, are found at the corresponding position in PIP5K1s, we postulate that the structural difference observed here may be related to the ability of the kinase to distinguish the two types of PIPK substrates.

### Kinase dimerization

**Figure 2:**
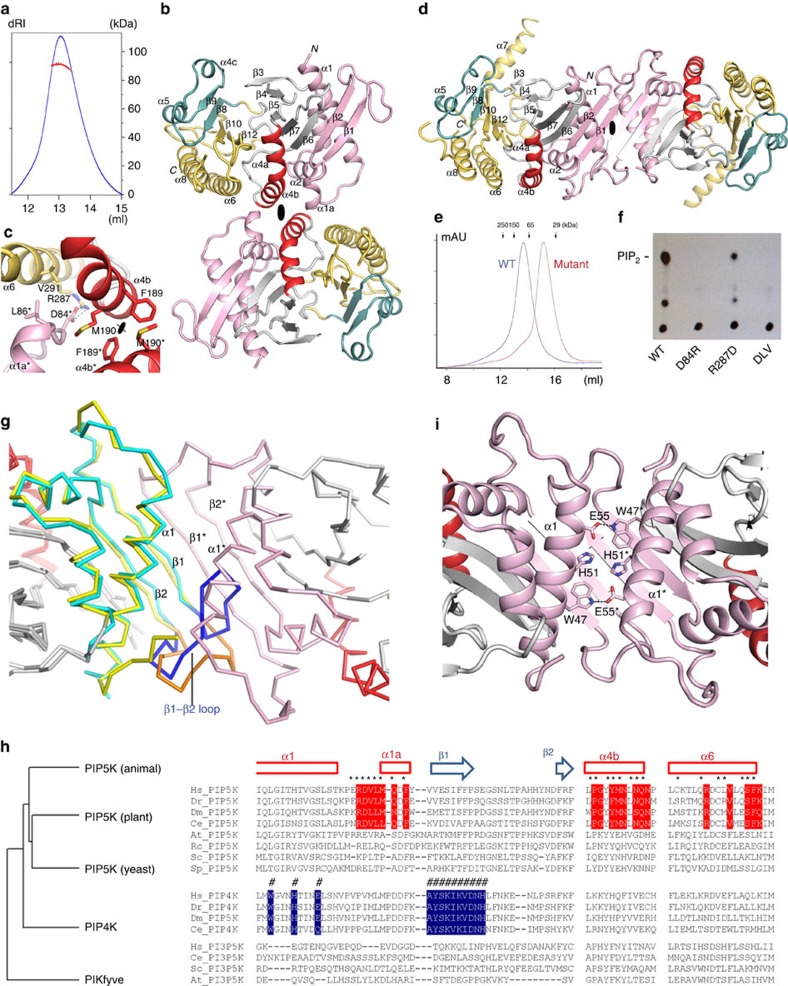
Dimerization of PIP5K1A catalytic domain. (**a**) SEC/MALLS shows that zPIP5K1A is dimeric in the solution. dRI, differential refractive index (blue trace). The calculated molecular weight is indicated by the red dots. (**b**) The side-to-side dimerization of zPIP5K1A. (**c**) A detailed view of the interactions that stabilize the dimer. This image corresponds approximately to the same view as that in **b**. (**d**) The head-to-head dimerization of PIP4K2B (PDB: 1BO1). (**e**) SEC elution profiles of the wild-type zPIP5K1A protein (blue) and its monomeric mutant DLV (brown). (**f**) Basal lipid kinase activity of the wild-type zPIP5K1A protein and its monomeric mutants. D84R, D84R mutant protein; PIP_2_, PtdIns4,5P_2_; R287D, D84R/R287D mutant protein; WT, wild-type protein. (**g**) Prevention of zPIP5K1A head-to-head dimerization by the β1–β2 loop. zPIP5K1A (cyan) is superimposed to one protomer of a PIP4K2A dimer (yellow, on the left). The β1–β2 loop of zPIP5K1A (blue) folds back, which is different from the fold of the corresponding region in PIP4K2B (orange), blocking dimerization in a head-to-head manner observed in PIP4K2B. The other protomer of the PIP4K2B dimer is shown in pink (right). (**h**) The phylogenetic relationship of type I (PIP5K), II (PIP4K) and III (PIKfyve) kinases. A multiple sequence alignment shows the conservation of residues at the dimeric interfaces of PIP5K (red) and PIP4K (blue). At, *Arabidopsis thaliana*; Ce, *Caenorhabditis elegans*; Dm, *Drosophila melanogaster*; Dr, *Danio rerio*; Hs, *Homo sapiens*; Rc, *Ricinus communis*; Sc, *Saccharomyces cerevisiae*; Sp, *Schizosaccharomyces pombe*. (**i**) Hydrogen bonds between the two α1 helices help to stabilize the PIP4K2B dimer.

The calculated molecular weight of PIP5K1A kinase domain is 44 kDa. Size-exclusion chromatography/multi-angle laser light scattering (SEC/MALLS) indicates that the kinase has a molecular weight of 90 kDa, suggesting that the protein is dimeric in the solution (Fig. 2a). The crystal structure reveals that the dimer has a side-to-side arrangement, involving both N- and C-lobes of the kinase (Fig. 2b). Most residues constituting the dimeric interface are found within three structural elements: the loop between α1 and α1a, α4b, and α6. α4b is near the centre of the dimer, and interacts with its symmetric mate (α4b*) through hydrophobic contacts mediated by Pro-186, Phe-189 and Met-190 (Fig. 2c). The interactions between the α1–α1a loop and α6, however, involve both charged and hydrophobic residues. The negatively charged Asp-84* from the α1*–α1a* loop is matched against a positively charged patch (Arg-287) on α6, while Leu-86* forms hydrophobic contact with Val-291. Dimerization buries a total of 1,850 Å^2^ protein surface area.

The dimerization mode of PIP5K1A is different from that of PIP4K2B (Fig. 2d). In PIP5K1A, both N- and C-terminal lobes contribute to dimerization, whereas in PIP4K2B, only the N-terminal lobes interact to form an elongated head-to-head dimer^20^. Despite this difference, many of the features for these kinases to interact with membrane surfaces are conserved. First, the angle of the two-fold axis relative to the monomer is nearly identical in both dimers (Supplementary Fig. 3a). Such a similarity cannot be coincidental, because the symmetry operation would enable the two active sites to open towards the same flat side of the dimer to simultaneously engage lipid head groups (Supplementary Fig. 3a). Second, the flat surface created by dimerization, 70 × 50 Å in PIP5K1A and 105 × 38 Å in PIP4K2B, is of comparable size (Supplementary Fig. 3b). And last, in PIP4K2B, the flat surface has a net positive charge (+14), which concentrates around the two-fold axis and forms a continuous patch. PIP5K1A carries less charge (+8), and its basic residues cluster around the active site, away from the centre of the dimer (Supplementary Fig. 3b). These positive charges are compatible with the close apposition of the kinase to the negatively charged membrane surfaces, and generate favourable electrostatic interactions^23^.

To confirm that the dimer observed in the crystal corresponds to the solution dimer, we introduced mutations to disrupt the dimerization. A triple D84R/L86A/V291A (DLV) mutation was first constructed to destabilize the packing between the α1–α1a loop and α6 by reversing the charge at position 84* and eliminating the hydrophobic interaction between Leu-86* and Val-291 (Fig. 2c). Gel filtration showed that the DLV mutant eluted significantly later than the wild-type protein, and SEC/MALLS confirmed that it was monomeric (Fig. 2e). Subsequent experiment showed that the D84R single mutant were also largely monomeric in solution. Both D84R and DLV mutants showed very low basal catalytic activity towards PtdIns4P in an *in vitro* kinase assay, demonstrating that dimerization may be important for enzyme function (Fig. 2f). To eliminate the possibility that the DLV monomeric mutant protein is misfolded, we compared the far-ultraviolet circular dichroism spectra of the wild-type and monomeric mutant proteins. As shown in Supplementary Fig. 4a, there is no fundamental difference between these two proteins, indicating the mutant protein is folded similarly to the wild-type protein. The subtle difference in the circular dichroism spectrum suggests that the structure of the DLV mutant protein may differ slightly from that of the wild-type protein and may explain the reduced basal kinase activity of the mutant. Importantly, introducing a second charge-reversal mutation (R287D) to restore the salt bridge at the dimeric interface between positions 84* and 287 clearly restored the activity of D84R single monomeric protein (Fig. 2f). As residues 84 and 287 do not interact with each other in a monomeric state, the ability of the second mutation at position 287 to restore the activity of D84R has to be achieved through protein dimerization. Collectively, our data strongly suggest that disrupting dimerization leads to a non-productive conformation, which can be rescued by the restoration of dimerization.

Amino-acid sequence analysis suggests that the two dimerization modes may be generally representative for the two types of PIPKs. The head-to-head mode of dimerization observed in PIP4K is prevented in PIP5K by the loop between β1 and β2, which folds back over β1 and clashes with the neighbouring α1* helix, when the PIP4K dimer is superimposed onto the PIP5K structure (Fig. 2g). Since the β1–β2 loop is not involved in crystallographic packing, its folded conformation most likely resembles that in the solution. The sequence of the β1–β2 loop in PIP5K differs from its counterpart in PIP4K and is longer by two amino acids (Fig. 2h). The side-to-side dimerization of PIP5K is stabilized by hydrophobic contact between the two α4b helices (Pro-186, Phe-189 and Met-190), and by favourable electrostatic interactions between Asp-84 (α1–α1a loop) and Arg-287 (α6) (Fig. 2c). Sequence alignment of type I (PIP5K), type II (PIP4K) and a more distant type III (PIKfyve) kinases shows that these features are present in all of the animal PIP5Ks, suggesting that they share the same mode of side-to-side dimerization (Fig. 2h). As the residues on the dimerization interface are largely conserved among different isoforms of animal PIP5Ks, it is conceivable that PIP5K isoforms may also form heterodimer. In plant and yeast PIP5Ks, however, the situation is less certain. Although their β1–β2 loops show high sequence homology with those in animal PIP5Ks, which exclude PIP4K-type dimerization, the hydrophobic residues on α4b and the ion pair from α1–α1a loop and α6 are not always present. Since the zebrafish PIP5K1A dimer can be readily disrupted by removing the ion pair, it is possible that plant and yeast PIP5Ks dimerize differently from animal PIP5Ks or exist as monomers. The PIP4Ks completely lack the sequence features described above. The head-to-head dimerization of human PIP4K2B is stabilized not only by forming an antiparallel β-sheet between the two β1 strands but also by a network of hydrogen bonds involving Trp-47, His-51 and Glu-55 between the two α1 helices (Fig. 2i). These residues are conserved among the PIP4Ks, consistent with the possibility that the head-to-head dimerization is a common feature for all of the type II kinases (Fig. 2h). The type III kinases (PIKfyve) lack all of the sequence features that are suggestive of either PIP5K-type or PIP4K-type dimerization. Different from type I and type II kinases, the type III kinases are much larger in size and have a complex domain structure, including an N-terminal FYVE finger domain, which binds PtdIns3P, the lipid substrate for type III kinase^24^.

### Interaction interface of PIP5K1A for DVL DIX

**Figure 3:**
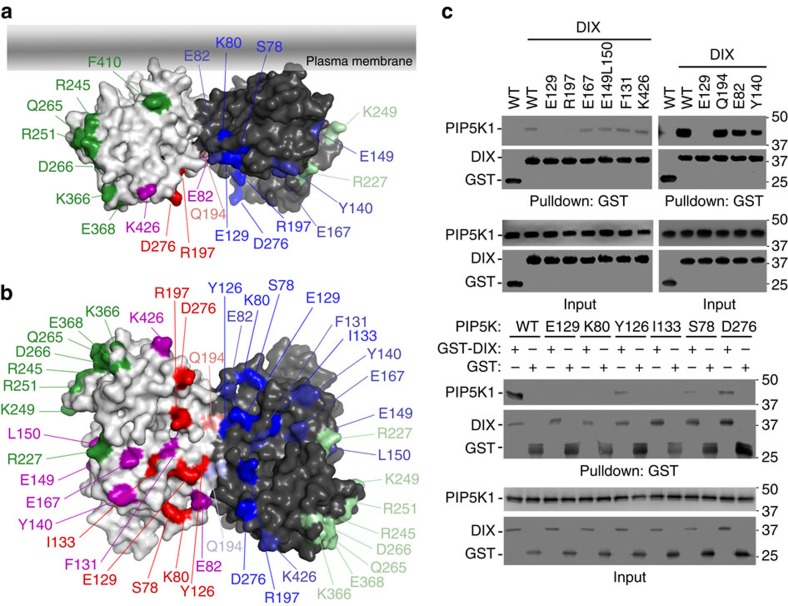
PIP5K1A interface for DIX interaction. (**a**,**b**) Schematic representation of mutated zPIP5K1A residues. Green marks those residues, when mutated, causing insolubility. Deep red and blue mark those critical for DIX binding. The rest do not affect the binding. (**b**) 90° Rotation of **a** with the membrane interaction interface at the back. (**c**) Interaction of recombinant zPIP5K1A and its mutants with recombinant DIX. GST-pull-down was performed with His-tagged zPIP5K1A proteins and GST-tagged DIX with GST as a control. The proteins were detected by western blotting. WT, wild type.

We have previously shown that the DIX domain of DVL directly bound to and stimulated the lipid kinase activity of mammalian PIP5K1 kinases^14^,^15^. To delineate the interaction interface for DVL DIX, we mutated zPIP5K1A residues as illustrated in Fig. 3a,b. These residues were selected for their conservation among the species (human, rodents, Xenopus and zebrafish) and exposure on the surface of the PIP5K dimer docked onto a membrane plane (Supplementary Fig. 3a). In a pull-down assay, mutation of residue Glu-129, Arg-197, Lys-80 or Ile-133 to Ala almost completely abrogated, whereas mutation of residue Tyr-126, Ser-78 or Asp-276 caused more than 70% reduction in the interaction with the recombinant DVL DIX protein (Fig. 3c). The DVL DIX protein used here is from mouse DVL1 (residues 1–106), containing a Y17D mutation to eliminate DIX aggregation^25^. By contrast, mutation of residue Glu-82, Phe-131, Try-140, Glu-149, Leu-150, Glu-167, Gln-194 or Lys-426 to Ala caused no more than 20% change in the interaction with DVL DIX (Fig. 3c). Mutation of residue Arg-227, Arg-245, Lys-249, Arg-251, Gln-265, Asp-266, Lys-366, Glu-368 or Phe-410 to Ala caused drastic reduction in protein solubility possibly due to mis-folding. Of note, most of these residues that cause insolubility when mutated are located in or near the substructure (cyan in Fig. 1a) unique to the PIPKs. These mutant proteins were not further analysed biochemically. Nevertheless, a DIX interaction interface composed of residues Glu-129, Arg-197, Lys-80, Leu-133, Tyr-126, Ser-78 and Asp-276 from both protomers was delineated on the PIP5K1A dimer, and this interface is located at the opposite side of the membrane/substrate interface as illustrated in Fig. 3a.

### DVL interaction in kinase activation and Wnt signalling

**Figure 4:**
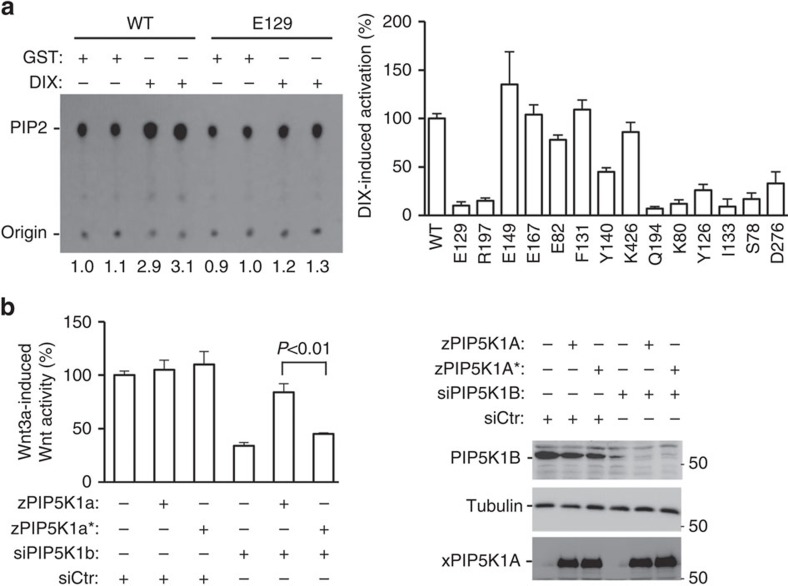
Regulation of PIP5K1A1 by DIX. (**a**) Regulation of the lipid kinase activity of zPIP5K1A by DIX. *In vitro* lipid kinase assay was performed using recombinant zPIP5K1A or its mutant proteins in the presence or absence of recombinant DIX proteins. Phospholipids were separated on thin-layer chromatography (TLC) plates (a representative TLC image is shown) and quantified by a phosphoimager. Dvl-induced lipid kinase activity of wild-type (WT) PIP5K1a was taken as 100%. Error bars stands for standard errors. The assays were repeated at least twice. (**b**) Expression of wild-type, but not E129A, zPIP5K1A rescues the inhibition of Wnt3a-induced reporter gene activity by PIP5K1A knockdown in HEK293 cells. Data are presented as means±s.d. (Student’s *t*-test, *n*=3). Protein expression was examined by western analysis.

Next, we examined the PIP5K1 mutants for their ability to be stimulated by DVL DIX using an *in vitro* kinase assay. Recombinant DIX protein was able to stimulate the lipid kinase activity of wild-type zPIP5K1A (Fig. 4a) as previously shown for mammalian PIP5K1 (ref. 15). By contrast, DIX-stimulated lipid kinase activity was markedly compromised for the E129A mutant that does not interact with DIX (Fig. 4a). Because the Glu-129 mutation did not reduce the basal lipid kinase activity (Fig. 4a), it is unlikely that the mutation resulted in significant conformational alterations in the catalytic core or membrane/substrate interface that are located at the other side of this residue. We also examined other zPIP5K1A mutants and observed a correlation between the interaction of the kinase mutants with DIX and their activation by DIX. While the zPIP5K1 mutants, including the E82A, F131A, Y140A, E149A, E167A and K426A mutants, which showed strong interactions with DIX, could still be stimulated by DIX, mutants, including R197A, S78A, K80A, Y126A, I133A and D276A, which showed compromised interactions with DIX, showed clear reduction in their activation by DIX (Fig. 4a). However, there is one notable exception to this correlation; the Q194A mutant was able to interact with zPIP5K1A (Fig. 3c), but unable to be strongly activated by DIX (Fig. 4a) (see below for discussion).

We also examined whether the interface between DIX and PIP5K1A characterized in this study was actually important for Wnt signalling. We have previously shown that knockdown of PIP5K1B in HEK293 cells led to a reduction in Wnt signalling^14^. This effect of PIP5K1B knockdown could be rescued by expression of wild-type zPIP5K1A, but not the E129A mutant (Fig. 4b). This result hence indicates that the interaction between DVL DIX and PIP5K1A has an important role in Wnt signal transduction.

### Regulation of PIP5K catalytic activity

**Figure 5:**
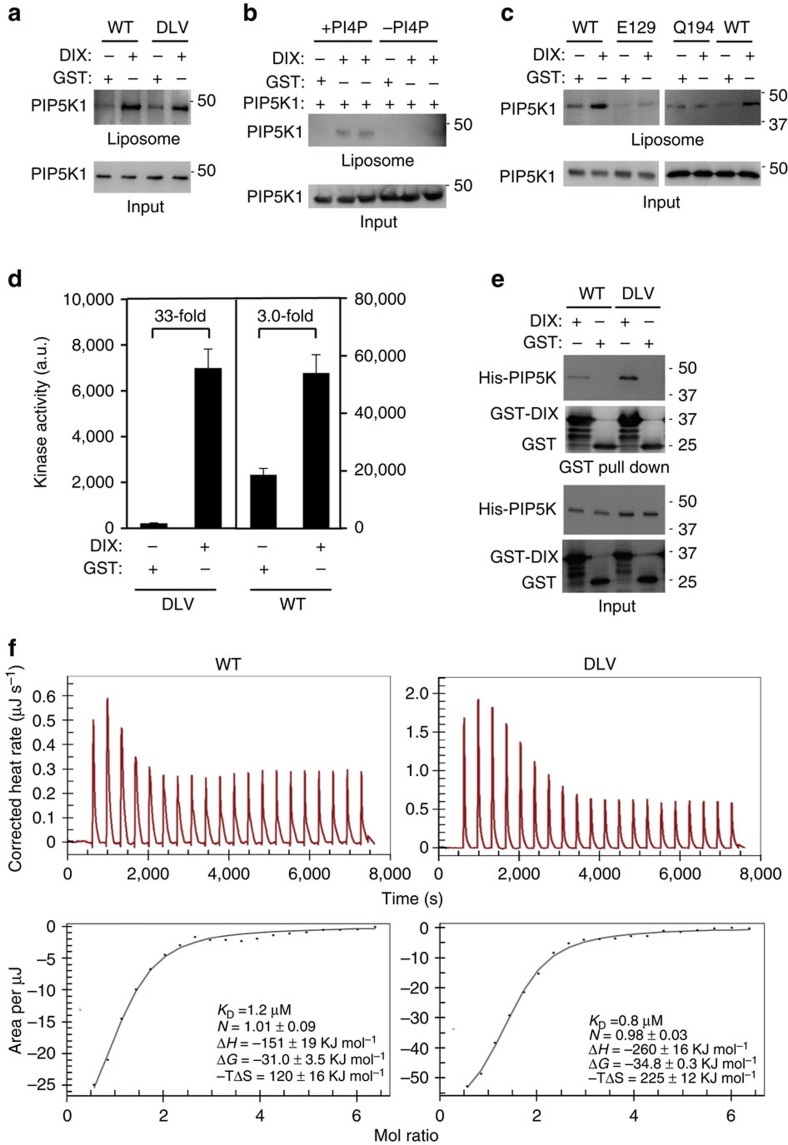
Regulation of zPIP5K1A enzymatic activity. (**a**) Binding of zPIP5K1A (wild type (WT)) and its monomeric DLV mutant to PtdIns4P-containing liposomes measured by using a liposome floatation assay in the presence of DIX or GST. (**b**) DIX stimulates PIP5K1 binding only to liposomes containing PtdIns4P. The liposome floatation assay was performed using phosphatidylcholine/phosphatidylserine liposomes with or without PtdIns4P. (**c**) Effect of the E129A and Q194A mutations on Dvl-induced binding of zPIP5K1A to PtdIns4P-containing liposomes. (**d**) Effects of DIX on the kinase activity of zPIP5K1A (WT) and its monomeric DLV mutant. Data are presented as means±s.d. (**e**) Interaction of zPIP5K1A (WT) and its monomeric DLV mutant with DIX. GST-pull-down was performed with His-tagged zPIP5K1A proteins and GST-tagged DIX with GST as a control. The proteins were detected by western blotting. (**f**) Assessment of the binding affinities of zPIP5K1A (WT) and its monomeric DLV mutant for DIX using ITC. The experiments were repeated twice times. The results from one of the experiments are shown.

Because dimerization increases the positively charged surface area (Supplementary Fig. 3b), the wild-type PIP5K1 dimer is presumably more favourable for binding to lipid membranes, where the kinase substrate resides, than its monomeric DLV mutant. We thus tested this assumption by comparing the wild-type zPIP5K1A molecule and its DLV mutant for their interactions with liposomes containing phosphatidylcholine/phosphatidylserine/PtdIns4P, which resembles the substrate environment in cells, using a liposome floatation assay^26^,^27^. Contrary to the above assumption, the dimeric wild-type and monomeric mutant molecules showed similar binding to the PtdIns4P-containing liposomes (Fig. 5a). Thus, the dimerization may not provide obvious advantage for the wild-type molecule to bind to lipid membranes or the substrate. We additionally found that DIX was able to stimulate the binding of zPIP5K1A and its DLV mutant to the liposomes (Fig. 5a). Of note, DIX was only able to stimulate the binding to liposomes containing PtdIns4P (Fig. 5b). By contrast, DIX failed to stimulate the binding of the E129A or Q194 mutant, whose kinase activity could not be stimulated by DIX, to the PtdIns4P-containing liposomes (Fig. 5c). These data suggest that, while the marked difference in the basal activity of the dimeric wild-type PIP5K1 and monomeric DLV mutant is very unlikely due to their ability to bind to the lipid membranes or substrate, stimulation of PIP5K1 binding to its substrate by DIX may be one of the mechanisms for DVL to activate the kinase. Consistently with this conclusion, the full-length DVL protein reduces the *K*_m_ of human PIP5K1B by sixfold (Supplementary Fig. 5).

Because DIX could stimulate the binding of the DLV mutant to PtdIns4P-containing liposomes, we anticipated that DIX should also be able to bind to and stimulate the kinase activity of the mutant. Despite its low basal activity, the DLV mutant was still capable of being activated by DIX. In fact, the magnitude of the activation of this mutant by DIX is far greater than that of the wild-type molecule; DIX activated the DLV mutant by more than 30-fold, whereas it activated the wild-type molecule by merely 3-fold (Fig. 5d). In addition, the DLV mutant appeared to show a stronger interaction with DIX than the wild-type protein in a pull-down assay (Fig. 5e). The assessment of these interactions by the isothermal titration calorimetry (ITC) experiment also suggests that the DLV mutant may have a higher affinity of DIX than wild-type PIP5K1 (Fig. 5f). However, this difference in the affinity for DXI between wild-type and DLV would not explain the much stronger activation of DLV by DIX than that of the wild type. Together with the similar ability for DIX to stimulate the wild-type and mutant PIP5K1 to bind to the liposomes containing PtdIns4P, it is more likely that the effect of DIX binding to the monomeric kinase on the regulation of the kinase activity differs from the effect of its binding to the dimeric kinase. In addition to its enhancement on substrate binding, DIX interaction with the monomeric mutant may in part act like the interaction occurred in PIP5K1 dimerization and stimulate the catalytic activity of the lipid kinase. It should be noted that, although the DLV mutant can be greatly activated by DIX, the DIX-stimulated activity is still much lower than the basal kinase activity of wild-type enzyme in the absence of DIX (Fig. 5d). It suggests that DIX interaction cannot fully substitute the function of dimerization in stimulating enzymatic activity.

## Discussion

In this work, we present the first crystal structure of a PIP5K1 molecule. Comparison of it with the prototypical protein kinase structure of PKA and the structure of a PIP4K2B has revealed important insights into the molecular characteristics of PIP5K1. The PIP5K1 structure also guided us to gain an understanding of the molecular basis for the regulation of this type of the lipid kinases by Dvl and dimerization. In addition, this study reaffirms the conclusion that DIX activates PIP5K1A via its direct interaction with the kinase, because all of the PIP5K1A mutants including E129A, R197A, K80A, I133A, Y126A, S78A and D276A that show compromised interactions with DIX show reduced activation by DIX. Furthermore, the failure to restore Wnt signalling activity by the E129A mutant in cells where PIP5K1 was silenced by siRNA (Fig. 4b) further supports the importance of DVL–PIP5K1 interaction in the Wnt signalling.

PIP5K1A and PIP4K2B share the same catalytic residues and signature motifs essential in their catalytic activity and low, but substantial, homology in their kinase domains (up to 33% identity in amino-acid sequence). Thus, it is reasonable to postulate that they may have the common origin in evolution. Our study also revealed similarities and differences between these two families of kinases. Unfortunately, the specificity loop (zPIP5K1A residues 386–401), in which the primary determinant of substrate specificity lies^28^, is disordered in the PIP5K1A structure as in the PIP4K2B structure (Rao *et al*.^20^), and therefore it remains difficult to rationalize the preferences of PIPKs towards different lipid substrates. However, the spatial proximity of the specificity loop to the subdomain unique to PIPKs raises the possibility that the two structural elements may function collaboratively to influence substrate binding to type I and type II PIPKs. Although the dimer arrangement and the distribution of positive charges differ widely between these two kinases, the orientation of the active site relative to the flat membrane-association surface, which is a fundamental and common feature for interfacial catalysis, is conserved.

One striking difference between PIP5K1A and PIP4K2B is the orientation and interface of dimerization. According to structure-based sequence alignment (Fig. 2h and Supplementary Fig. 2), PIP4Ks across species are very likely to dimerize in a head-to-head manner, because the key residues on dimerization interface observed in the PIP4K2B structure are highly conserved. On the contrary, it appears that only animal PIP5Ks may dimerize in the side-to-side way. Because the yeast PIP5K, which is a prototype of this family of kinases^18^,^29^ and plant PIP5Ks show low amino-acid sequence homology with zebrafish PIP5K1A or human PIP4K2B at the dimerization interfaces, these PIP5Ks may not dimer at all or oligomerize in a different way. The side-to-side dimerization of animal PIP5Ks may be a feature evolved at or after the divergent point during evolution.

Dimerization could provide a means to increase the binding affinity of the kinase to the membrane by doubling the basic surface area that contacts the acidic lipid head groups, and by creating a flat surface that is complementary in shape to the membrane plane. Alteration of the charges on this flat charged surface in PIP4K2B was shown to diminish membrane binding and kinase activity^20^,^23^. However, the monomeric PIP5K1A mutant, despite its low basal kinase activity, appears to bind to PtdIns4P-containing liposomes similarly to the dimeric wild-type molecule (Fig. 5a). This result suggests membrane or substrate binding may not be the key reason by which the dimer has a higher kinase activity. Dimerization of PIP5K1A may instead play a structural role in keeping the kinase in the productive conformation. Because the DLV monomeric zPIP5K1A mutant appears to be less stable than the dimeric wild-type kinase (Supplementary Fig. 6), dimerization may contribute to the protein stability. However, the difference in the protein stability would not explain the drastic difference in the kinase activity between the dimeric and monomeric kinase. Thus, dimerization has to contribute in other ways. In PIP5K1A dimer, α4b, which immediately follows α4a (‘C helix’ in protein kinases), tightly packs against α4b* from the other protomer at the centre of the dimerization interface. It is conceivable that the tension applied on α4b is released in the monomer, resulting in conformational changes of α4b, which may be transmitted to α4a through the long and bent helix (α4a plus α4b). As ‘C helix’ is a known element crucial for kinase activity regulation^21^, the conformational changes of α4a brought about by monomer-to-dimer transition may also contributes to the increase in the kinase activity in the dimeric enzyme. This mechanism of dimerization-mediated activation of enzymatic activity may be used to explain the observation of the far greater activation of monomeric PIP5K1 mutant than the dimeric wild-type enzyme by DIX (Fig. 5d). The binding of DIX to the monomeric molecule may partially resemble the dimerization interaction to stimulate the kinase activity. In fact, the dimerization and DIX interaction interfaces are adjacent (Fig. 3a). Together with the location of R197, a zPIP5K1 residue critical for DIX binding, at the loop connecting to the α4a helix, it is reasonable to postulate that DIX binding may trigger changes in the kinase structure similar to what dimerization induces, which eventually leads to an increase in the catalytic activity.

In addition to its role in stimulation of the catalytic activity of monomeric PIP5K1, DIX binding has another role in stimulation of PIP5K enzymatic activity; DIX binding to PIP5K1 enhances the binding of PIP5K1 to its substrate PtdIns4P (Fig. 5a–c). This role of DIX may not be shared by dimerization-mediated interaction, because DIX stimulates both dimeric and monomeric kinase similarly in this regard (Fig. 5a). Because PIP5K1 is likely to exist as a dimer in cells, this role of DIX binding may be more relevant to its stimulation of PIP5K1 activity in cells. Given that the DIX interaction interface and the substrate-binding site are at opposite sides of the PIP5K1 molecule, DIX binding at one side may have to enhance substrate binding at the opposite side though a conformational change. The residue Q194 may have a key role in transducing such conformational change. Although mutation of Q194 did not obviously affect its basal kinase activity or interaction with DIX (Fig. 3c), DIX failed to stimulate either kinase activity (Fig. 4a) or substrate binding (Fig. 5c) of this mutant. Given Q194 is located at the end of the α4b helix, it is conceivable that the helix is also involved in the transduction of the change to the substrate interface on DIX binding. In addition to the enhancement of substrate binding, DIX may also stimulate the dimeric wild-type kinase by stimulating its catalytic activity via a mechanism described earlier for the monomeric kinase molecule. This conclusion can be extended to the activation of human PIP5K1 by DVL, because the full-length DVL3 protein increases the *V*_max_ , while reducing the *K*_m_ of human PIP5K1B (Supplementary Fig. 5). In other words, DVL3 increases both the catalytic activity and substrate affinity of human PIP5K1B.

The importance of this DIX interaction interface in regulation of the PIP5K1A kinase activity revealed by this study may provide a paradigm for the understanding of the regulation of the PIP5K1 family of kinases by other regulators via protein–protein interactions. The PIP5K1 family of kinases are also regulated by their interactions with Talin^30^,^31^, RAC1 (refs 32, 33, 34), ARF6 (refs 35, 36), ARF1 (ref. 37) and the adaptin-2 complex^38^,^39^,^40^,^41^. These interactions underlie the roles of PtdIns4,5P2 in a wide range of cellular activities including cell migration, adhesion and endocytosis. Of note, RAC1, ARFs and the adaptin-2 complex have been shown to directly stimulate the lipid kinase activity. In particular, mouse PIP5K1B residue Glu-61, which corresponds to Glu-82 of zPIP5K1A, was shown to be involved in the interaction with RAC1 (ref. 33). This residue is located at the vicinity of the DIX-binding interface, even though it is not directly involved in DIX binding (Fig. 3). Thus, RAC may activates PIP5K1 with a mechanisms similar to that of DIX. Further study is warranted to test the idea.

## Methods

### Protein preparation

The DNA sequence encoding the kinase catalytic domain of zebrafish PIP5K1Aα (residues 49–431) was amplified by PCR from cDNA and inserted into a modified pET21b expression vector (Novagen) at NheI and XhoI sites. Accordingly, the recombinant protein has an extra N-terminal sequence of ‘Met-Ala-Ser’ and a C-terminal sequence of ‘Leu-Glu-His_6_’. The protein was overexpressed in BL21-CodonPlus(DE3)-RIL cells (Stratagene) in Luria–Bertani medium. The cells were induced with 0.1 mM isopropyl-b-D-thiogalactoside at an OD_600_ of 0.4 and were left to grow overnight at room temperature. The cells were collected and re-suspended in the lysis buffer of 50 mM HEPES pH 7.3, 300 mM NaCl, 5% glycerol, 0.5% Triton X-100 (American Bioanalytical) and EDTA-free protease inhibitor cocktail (Roche). After lysis by sonication, the cell lysate was applied to high-speed centrifugation (20,000*g* at 4 °C) for 30 min and the resulting supernatant was loaded onto a Talon metal affinity column (Clontech). The recombinant protein was eluted with 200 mM imidazole in a washing buffer containing 20 mM HEPES (pH 7.3), 300 mM NaCl, 5% glycerol and 0.03% Triton X-100. The protein was concentrated and further purified on a Superdex S-200 column (GE Healthcare) in a chromatographic buffer containing 10 mM HEPES (pH 7.3), 300 mM NaCl, 5% glycerol and 0.01% Triton X-100. The peak fraction was pooled for crystallization or other studies. All mutations were generated using the Quikchange site-directed mutagenesis kit, and the mutant proteins were expressed and purified in the same way as the wild-type protein.

The DIX domains (1–106 and 1–200; the latter referred to as DIX-L) of mouse DVL1 with a substitution of Asp for Tyr-17 were expressed in *Escherichia coli* as glutathione S-transferase (GST) fusion proteins as described above. The recombinant proteins were prepared after affinity purification using glutathione–agarose beads followed by size-exclusion chromatography using an FPLC.

### Crystallization and structural determination

Purified zebrafish PIP5K1A was concentrated to 5 mg ml^−1^ to be used for crystallization screening at 4 °C using a sitting drop format. A single-crystallization lead was identified in 0.1 M MES pH 6.5, 12% PEG 20000. The tiny (10 μm in diagonal) and pyramid-shaped crystals were difficult to reproduce, and diffracted only to 8 Å resolution at beamline X29A at National Synchrotron Light Source. In an extensive additive screen, spermidine (Sigma-Aldrich) was found to improve reproducibility, increasing the size of the crystals to 50 μm. The crystals were cryo-protected in 25% glycerol in 0.1 M MES pH 6.5, 9% PEG 20,000 and flash frozen in liquid nitrogen. At X29A, these bigger crystals diffracted to 3.3 Å resolution. Decay in diffraction quality due to radiation damage was observed during data collection, and only the first 120 frames from the two best data sets were merged. Diffraction data were indexed and scaled using *HKL2000* (ref. 42). Molecular replacement was performed by using Auto-MR in *PHENIX*^43^. The A chain of hPIP4K (PDB entry 1BO1) was used as the search model. Since the electron density was poor and the map covering the kinase’s C-lobe was not interpretable, we modified the initial approach by using the N- and C-lobes of hPIP4K as separate search models in molecular replacement, and by treating them with CHAINSAW in *CCP4i* to remove segments with large divergence in primary sequence^44^. The search models were further improved by manual deletion of segments with poor densities. Iterated cycles of model building and refinement were performed using *COOT*^45^, *PHENIX* and REFMAC5 as implemented in *CCP4i*. TLS refinement at the last stage of refinement further improved the electron density map.

### PIP5K1A kinase assay

The *in vitro* lipid kinase assay was performed as reported (Pan *et al*.^14^) and detailed as the following. In 50-μl reaction buffer, which contains 100 mM Tris-HCl (pH 7.4), 50 mM EGTA, 100 mM MgCl_2_, 10 ng purified enzyme was incubated with 100 ng DIX, 10 μCi [^33^P]-ATP, 50 μM cold ATP and 80 μM PI(4)P (A.G. Scientific) at room temperature for 30 min. The reaction was stopped by adding the lipid extraction solution, which contains chloroform, methanol and HCl with a volume ratio of 3.3:3.7:0.1, and 10 μg per ml brain extract from bovine brain (BFF). After vortexing for 1 min, the sample was centrifuged at 15,000*g* for 2 min at room temperature, and the lower organic phase was collected and dried under vacuum. The dried lipids were solubilized in the sample solution, a 2:1 mixture (by volume) of chloroform and methanol, and separated by thin-layer chromatography. The product of the reaction, PI(4,5)P_2_, was visualized and quantified by a phosphorimager.

### SEC/MALLS

Data were collected from a Superdex S-200 10/30 HR column (GE Healthcare) connected to the FPLC System. Elution from the column was monitored by a photodiode array ultraviolet/visible detector (2996 PDA, Waters Corp.), differential refractometer (OptiLab-rEx Wyatt Corp.), and static, multi-angle laser light-scattering detector (DAWN-EOS, Wyatt Corp.). The system was equilibrated with a running buffer containing 10 mM HEPES (pH 7.3), 300 mM NaCl, 5% glycerol and 0.01% TX-100 at a flow rate of 0.5 ml min^−1^. Average molecular weight was determined by *ASTRA* that uses a Rayleigh–Debye–Gans light-scattering model, relating the amount of scattered light to the concentration and molecular weight of the solute and second virial coefficient:




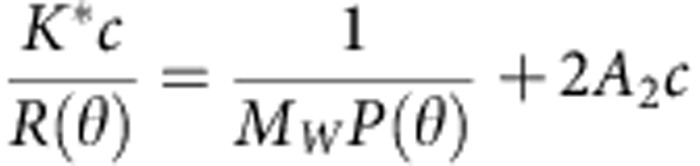




where *R*(*θ*) is the intensity of excess scattered light at angle *θ*; *c* is the concentration of the solute; *M*_*W*_ is the weight average molecular weight of the solute; *A*_2_ is the second virial coefficient; *P*(*θ*) describes the angular dependence of the scattered light; K* is an optical parameter equal to 4π^2^n^2^(d*n*/d*c*)^2^/(*λ*_0_^4^*N*_A_); *n* is the refractive index; d*n*/d*c* is the refractive index increment for the solute; *N*_A_ is Avogadro’s number; and *λ*_0_ is the wavelength of the scattered light^46^.

### Circular dichroism spectrum

The purified protein was diluted to 0.25 mg ml^−1^ using gel filtration buffer (10 mM HEPES (pH 7.3), 300 mM NaCl, 5% glycerol and 0.01% Triton X-100) and the circular dichroism spectra were recorded on a Chirascan circular dichroism spectrometer (Applied Photophysics) from 190 to 260 nm at 15°. For each sample, three scans were performed and averaged. Owing to the high salt concentration (required for sample stability), the signals at the very far-ultraviolet region (190–204 nm) are very noisy and therefore not included for analysis.

### Stability assay

To evaluate the stability of the enzyme, the protein purified on ice was diluted to 0.1 mg ml^−1^ using the cold gel filtration buffer (10 mM HEPES (pH 7.3), 300 mM NaCl, 5% glycerol and 0.01% Triton X-100) and then incubated at 37° for 30 min before being put back on ice. The activities of the treated samples were measured as mentioned above. To accurately measure the very low activity of the DLV mutant protein, 300 ng enzyme was added, 30 times higher than the wild-type protein in kinase assay. Three replicates were performed for each sample.

### Pull-down assay

Recombinant proteins (0.5 μg PIP5K1 and 0.5 μg GST-DVL or GST) were incubated in 200 μl of the binding buffer (10 mM HEPES (pH 7.4), 150 mM NaCl, 0.2% Triton, 0.01% SDS, 3 mM dithiothreitol, 10% glycerol and 1 × protease inhibitor) at 4 °C for overnight. Glutathione beads pre-blocked with 1% BSA were then added for 2 h. After the beads were washed five times with the binding buffer, the pull-down complexes were analysed by western blotting. Uncropped images for some of the important blots are shown in Supplementary Fig. 7.

### Liposome flotation assay

Phosphatidylinositol lipids (87.8 mol% phosphatidylcholine and 8.8 mol% phosphatidylserine in the presence of 3.4 mol% PtdIns4P or 3.4 mol% PtdIns; purchased from Avanti Polar Lipids) were dissolved in a chloroform:methanol mixture. The solvent was then evaporated using nitrogen stream in a fume hood, yielding a thin lipid film. Dried lipid was added with the lipid reconstitution buffer (25 mM HEPES, pH 7.4, 100 mM KCl, 10% glyceril and 1% dithiothreitol) and vortexed vigorously for 5 min, followed by repeated freeze thaw (seven times) using liquid nitrogen and hand warming. The lipid suspension was sonicated for 10 min in a bath sonicator. The liposomes were incubated with recombinant proteins (1 2 μg PIP5K, 2.47 μg DIX or 1.67 μg) for 30 min on ice in 75 μl of 25 mM HEPES, pH 7.4, 100 mM KCl, 10% glycerol and 1 mM dithiothreitol in the presence of 100 μM of free Ca^2+^. A volume of 75 μl of 80% Accudenz was added to the liposome–protein mixture to yield a final concentration of 40% Accudenz. A total of 30% Accudenz was then layered on the top, and the samples were centrifuged for 4 h in a TLA100 rotor at 32,000 r.p.m. The floated fraction and inputs were analysed by western blotting.

### Wnt activity assay

The Wnt reporter gene assay was performed in HEK293 cells (American Typer Culture Collection). The cells were first transfected with siRNAs using RNAiMax followed by another transfection of Wnt reporter genes (*TOPFlash* and *GFP*) and siRNAs using Lipofectamine Plus 2 days later. Luciferase assays were performed 24 h after the second transfection and 6 h after Wnt3a stimulation. Luciferase activity was normalized against the fluorescence intensity of coexpressed GFP.

### Isothermal calorimetry assay

ITC was performed in a Nano ITC Low Volume instrument (TA Instruments). wild-type PIP5K or its DLV mutant (0.005 mM) was prepared in a buffer (10 mM HEPES (pH 7.3), 300 mM NaCl, 5% glycerol and 0.04% Triton X-100) and placed in an ITC sample cell with a reaction volume of 182 μl. Dix109 (0.1 mM) was prepared in the same buffer and added into the sample cell via sequential injection (2.5 μl per injection) at an interval of 350 s under constant stirring (250 r.p.m.).

The thermograms were applied for baseline correction. A blank correction for the heat of dilution was made by subtracting the integrated peak area from a constant estimated from the convergence value of the final injections. The corrected heat change (Δ*Q*) was plotted against the molar ratio of Dix109/wild-type PIP5K or Dix109/TM-PIP5K. Values for binding stoichiometry (*n*), the dissociation constant (*K*_D_), enthalpy change (Δ*H*) and entropies change (Δ*S*) were directly determined by fitting the data using the one-site independent binding model provided by NanoAnalyze software (Version 3.3.0, TA Instruments). Δ*G* (Gibbs free energy change)=Δ*H*−*T*Δ*S*. *R*, gas constant, 8.315 J mol^−1^ K; *T*, absolute temperature.

## Additional information

**Accession codes**: The atomic coordinates and structure factors (accession code 4TZ7) have been deposited in the protein data bank, Research Collaboratory for Structural Bioinformatics, Rutgers University, New Brunswick, NJ (http://www.rcsb.org/).

**How to cite this article:** Hu, J. *et al*. Resolution of structure of PIP5K1A reveals molecular mechanism for its regulation by dimerization and dishevelled. *Nat. Commun.* 6:8205 doi: 10.1038/ncomms9205 (2015).

## Supplementary Material

Supplementary Information Supplementary Figures 1-7

## Figures and Tables

**Table 1:** Crystallographic statistics.

**Data collection**	
Wavelength (Å)	1.075
Space group	P43212
Cell dimensions (Å)	*a*=*b*=88.9, *c*=157.1
Resolution (Å)*	40–3.3 (3.4–3.3)
Redundancy*	13.6 (14.4)
Completeness (%)*	99.5 (100)
<*I*/*σ*>*	30.1 (4.9)
*R*_merge_*†	0.075 (0.637)
	
**Refinement**	
Unique reflections	9,947
Number of atoms	2,295
*R*_work_/*R*_free_‡	0.212/0.265
Averaged B-factors (Å^2^)	52
R.m.s.d.	
Bond lengths (Å)	0.0073
Bond angles (°)	1.235

R.m.s.d, root-mean-squared deviation

^*^Highest-resolution shell is shown in parentheses.

^†^*R*_merge_=∑|I_*i*_−<I>|/∑I_*i*_

^‡^*R*_work_=∑|F_o_−F_c_|/∑F_o_. *R*_free_ is the cross-validation *R* factor for the test set of reflections (10% of the total) omitted in model refinement.
