# Supplementary material for: Resolution of structure of PIP5K1A reveals molecular mechanism for its regulation by dimerization and dishevelled
Source: Nat Commun. 2015 Sep 14;6:8205. doi: 10.1038/ncomms9205 (PMC4570271; doi:10.1038/ncomms9205)
Supplement: Supplementary Information — Supplementary Figures 1-7 [file ncomms9205-s1.pdf]

SUPPLEMENTARY INFORMATION:

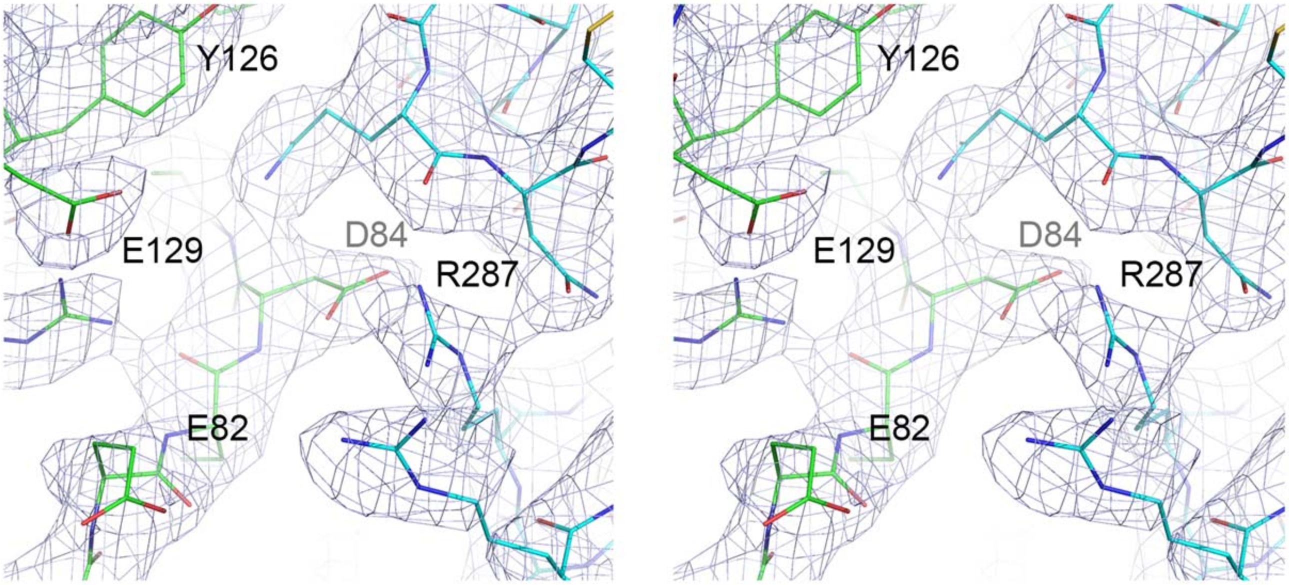

Supplementary Figure1. Cross-eyed stereo view of 2Fo-Fc electron density map (σ=1) at the dimerization and DVL interaction interfaces of PIP5K1A. Residues studied in this work are labeled

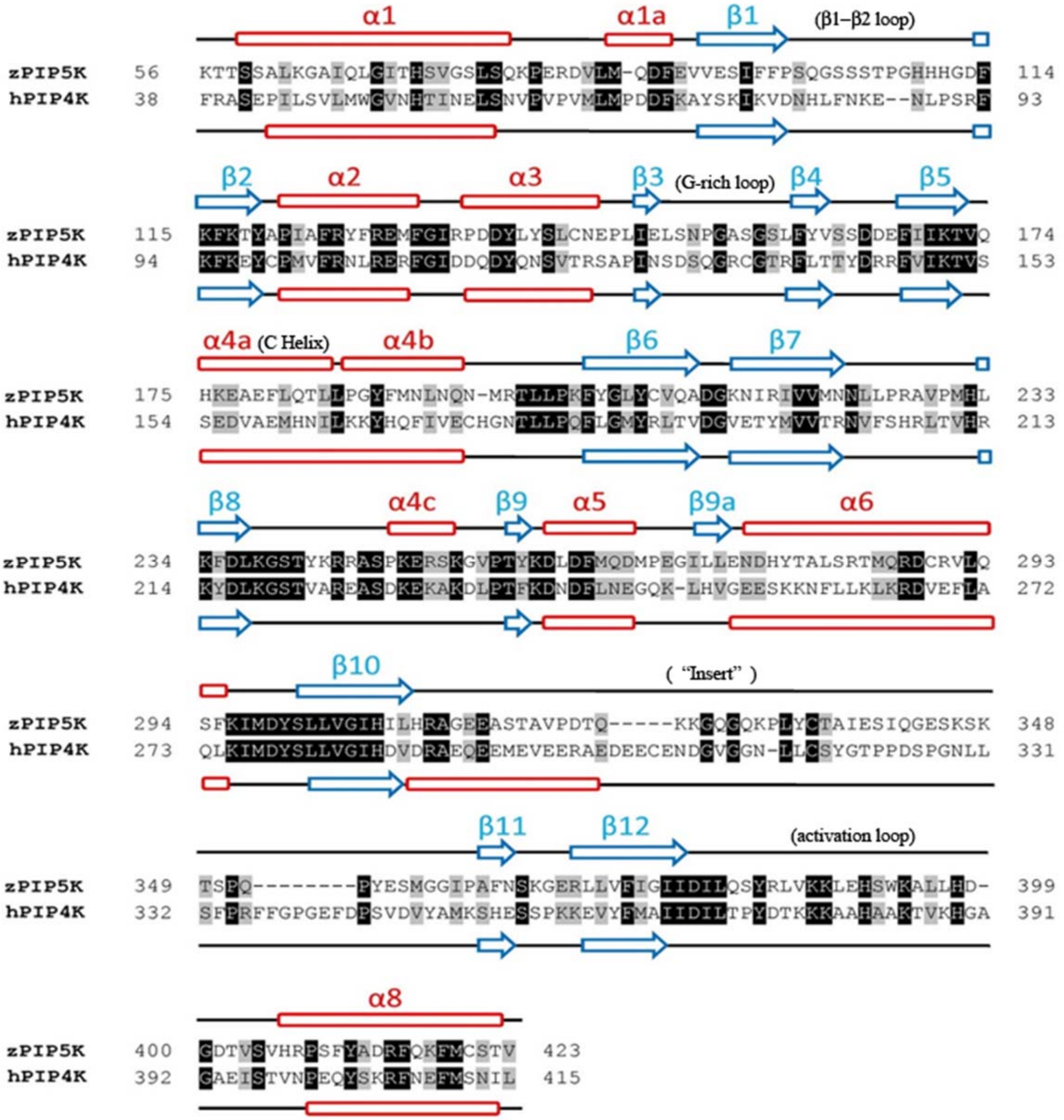

Supplementary Figure 2. Structure-based sequence alignment between zebrafish PIP5K1A and human PIP4K2B. The α-helices are indicated by the red boxes, and β-strands by the blue arrows. Identical residues are highlighted in black. Some important structural elements are indicated in brackets.

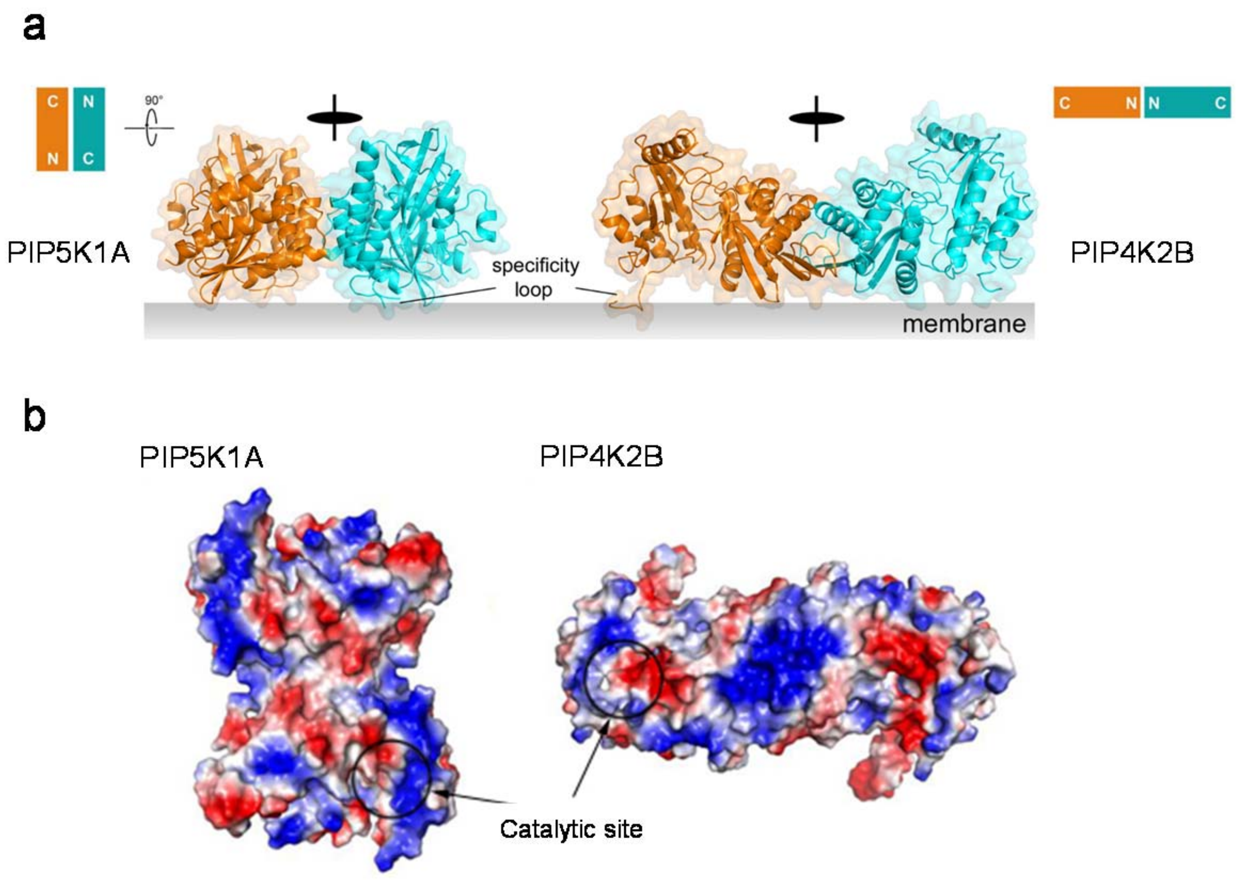

Supplementary Figure 3. Comparison of PIP5K1A dimerization to that of PIP4K2B.

a) A model of zPIP5K1A and PIP4K2B bound to the membrane surface (grey box).

b) The membrane-binding surfaces. The molecular surfaces are colored according to electrostatic potential (blue, positive; red, negative). The catalytic sites are denoted.

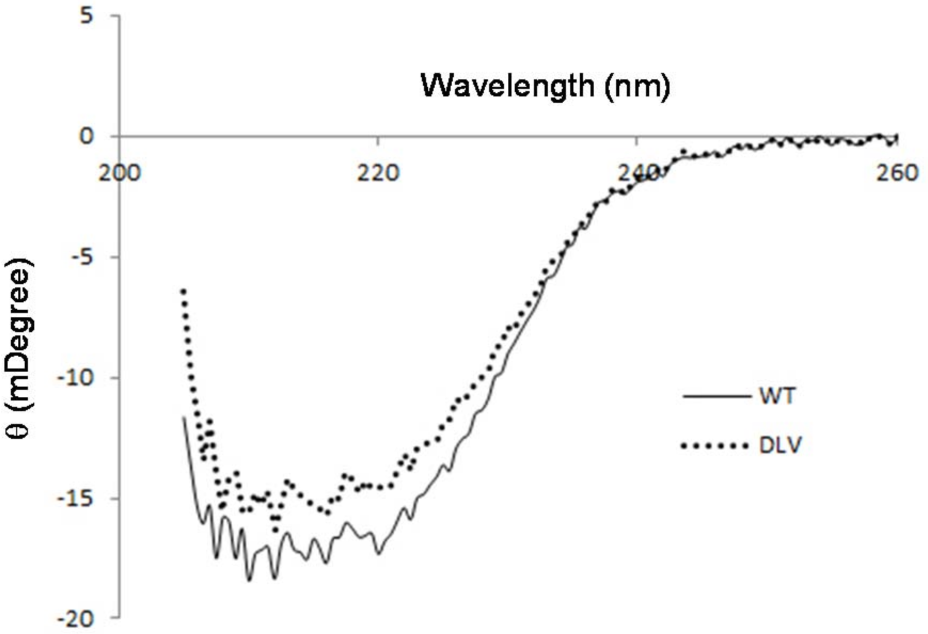

Supplementary Figure 4. Circular Dichroism spectra of wild type zPIP5K1A (solid line) and the DLV monomeric mutant protein (dotted line). Both proteins were in the gel filtration buffer (10 mM HEPES pH 7.3, 300 mM NaCl, 5% glycerol and 0.01% TX-100) and adjusted to the same concentration (0.25 mg/ml). The CD spectra were recorded at 15 degree from 190 nm to 260 nm. For clarity, the very noisy signals at the very far UV region (190 nm to 204 nm, due to high salt buffer) are not shown.

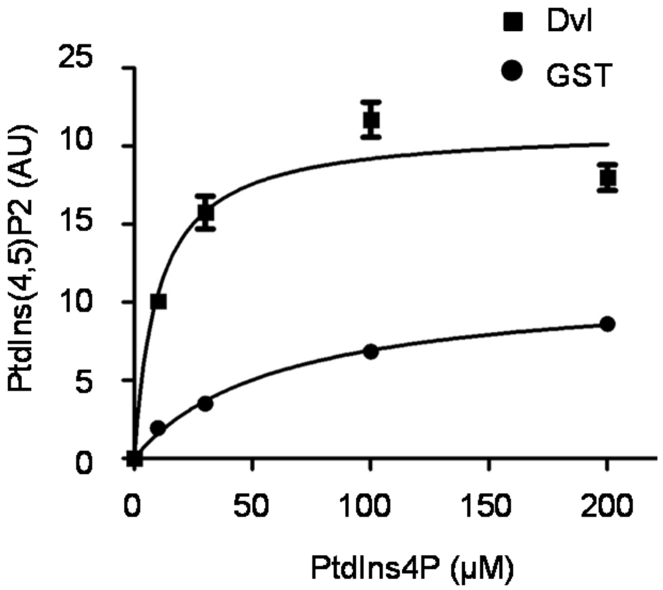

	GST	DVL
K_m (mM)	60.83±7.1	10.16±2.9
V_{max} (AU)	11.14±0.5	21.15±1.2

Supplementary Figure 5. Effects of Dvl on the kinetics of human PIP5K1B enzymatic activity. Lipid kinase activity of purified human PIP5K1B (50 nM) was determined by an in vitro kinase assay followed by TLC analysis in the presence of purified GST and Dvl3 proteins (100 nM). Values are presented as means±SD from three independent experiments ($P<0.05$ for K_m and V_{max} between GST and DVL, Student's t-Test).

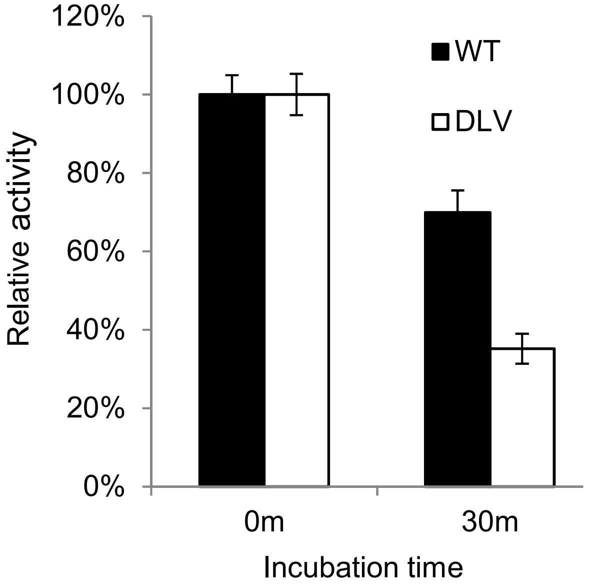

Supplementary Figure 6. Stability assay of wild type zPIP5K1A (filled column) and the DLV monomeric mutant protein (open column). The wild type protein and the mutant protein were first incubated at 37 degree, and then the kinase activities were measured. The relative activities were calculated by the ratio of the activity at each time point to the activity without treatment. The averages of three experiments are shown with standard deviations denoted by the error bars.

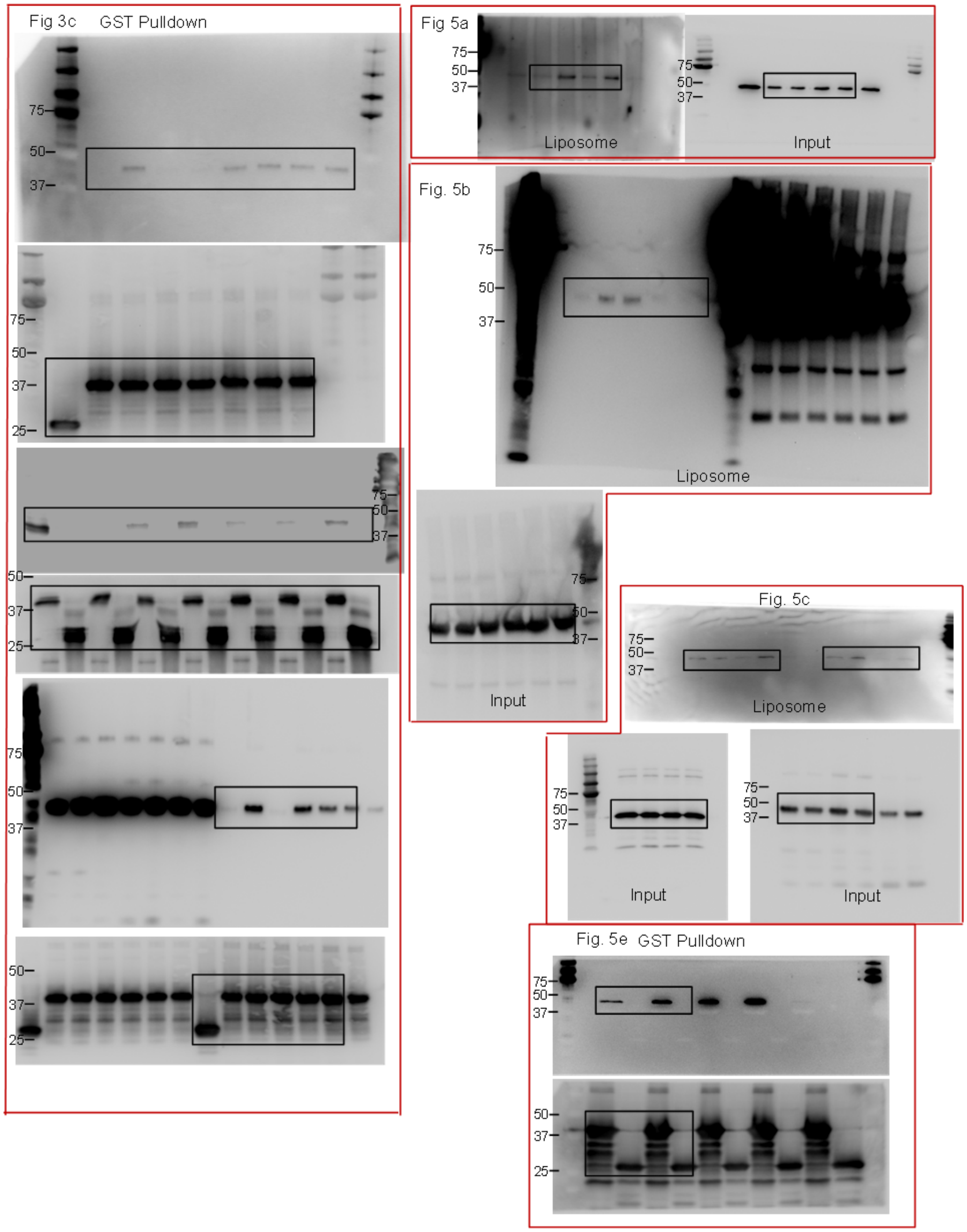

Supplementary Figure 7. Uncropped Western blots
